# Cross-cultural adaptation and psychometric properties study of Prolonged Grief Disorder Questionnaire (PG-12-R) for caregivers of terminal cancer patients, Thai version

**DOI:** 10.1371/journal.pone.0343917

**Published:** 2026-07-15

**Authors:** Apinya Sae-Lim, Chathaya Wongrathanandha, Thayakorn Kittichai, Arthit Chaithanasarn

**Affiliations:** 1 Department of Family Medicine, Faculty of Medicine Ramathibodi Hospital, Mahidol University, Bangkok, Thailand; 2 Department of Community Medicine, Faculty of Medicine Ramathibodi Hospital, Mahidol University, Bangkok, Thailand; Universiti Sains Malaysia - Kampus Kesihatan, MALAYSIA

## Abstract

**Objectives:**

To translate, culturally adapt, and validate a Thai version of the Prolonged Grief Disorder Questionnaire (PG-12-R) for application with caregivers of terminal cancer patients.

**Methods:**

This quantitative cross-sectional study was conducted in two phases. The translation and cultural adaptation processes were in accordance with established guidelines, which included forward translation, reconciliation, back translation, expert committee review, pilot testing with 30 caregivers, and finalization. The validation phase comprised 165 caregivers recruited from the palliative care service at Ramathibodi Hospital. Participants completed the Thai PG-12-R, Thai versions of the Generalized Anxiety Disorder–7 (GAD-7), the Patient Health Questionnaire–9 (PHQ-9), and the Short Form Zarit Burden Interview (ZBI). The test–retest reliability was assessed over a two-week interval. The psychometric evaluation included Cronbach’s alpha, intraclass correlation coefficients (ICC), content validity index (CVI), exploratory factor analysis (EFA), simple linear regression coefficient, and Spearman’s correlation coefficients.

**Results:**

The Thai version of the PG-12-R demonstrated excellent content validity (scale-level CVI = 0.92). Face validity ensured that the scale was culturally adapted to Thai expressions of grief. Structural validity assessment using EFA supported a unidimensional model (excluding Item Q3 due to low factor loading), with the single factor accounting for 67.61% of the total variance (eigenvalue = 4.27). The scale showed good internal consistency (Cronbach’s alpha = 0.87) and test–retest reliability (ICC = 0.88). Convergent validity was established through significant positive correlations with depression (PHQ-9: Spearman’s ρ = 0.67), anxiety (GAD-7: ρ = 0.59), and caregiver burden (ZBI: ρ = 0.60). Simple linear regression analysis confirmed that scores on the PHQ-9 (β=1.11), GAD-7 (β=1.20), and ZBI (β=0.54) were all significant predictors of grief severity.

**Conclusions:**

The modified Thai PG-12-R is a reliable and valid instrument for assessing anticipatory grief in caregivers of terminally ill cancer patients. Its conciseness and accessibility enable its application in conventional palliative care for early identification of high-risk caregivers and the development of specific interventions.

## Introduction

Cancer is a significant global public health concern, affecting millions of patients and their families. Globally, there were approximately 20 million new cancer cases and 9.7 million cancer-related deaths in 2022, with Thailand reporting an estimated 183,000 new cases and 118,000 deaths [1]. Additionally, the incidence of cancer tends to increase over time. This growing burden necessitates support systems for both patients’ needs and the mental and emotional health of their caregivers. Palliative care is crucial for individuals with advanced cancer as it aims to enhance their quality of life.

In recent years, awareness has increased regarding the emotional challenges faced by family members and caregivers, particularly concerning anticipatory grief—the emotional response to the impending loss of a loved one [2]. Since anticipatory grief takes place before death and involves complex emotional, cognitive, and behavioral responses, it is distinct from post-loss grief. Many caregivers of life-limiting illnesses are aware of this phenomenon, and some research suggests that its prevalence rates are higher than those of post-loss grief [3]. The prevalence of anticipatory grief among cancer patients’ relatives ranges from 14.9% to 33%, depending on the assessment scales used [4]. Elevated anticipatory grief is associated with decreased problem-solving and decision-making skills [5], increased anxiety and depression [6], and increased susceptibility to Prolonged Grief Disorder (PGD) after bereavement [7].

According to the International Classification of Diseases, 11th Revision (ICD-11), PGD is a mental disorder marked by persistent, intense longing for or preoccupation with the deceased, as well as functional impairment [8,9]. Compared to deaths from other causes, cancer-related deaths are a significant predictor of PGD, and caregivers of cancer patients are seen as a particularly high-risk group [10,11]. Addressing anticipatory grief before loss is an increasingly recognized way to help mitigate the risk of PGD. To identify caregivers at risk for significant anticipatory grief and plan appropriate interventions, reliable and valid assessment tools are needed.

Several instruments have been developed to assess anticipatory grief, including the Anticipatory Grief Scale (AGS) [12], the Prolonged Grief Disorder Questionnaire (PG-12) [13], the Marwit–Meuser Caregiver Grief Inventory (MM-CGI), and its abbreviated version (MM-CGI-SF) [14–16]. The MM-CGI has been validated for cancer patient caregivers; nonetheless, its length may impede practicality in clinical settings [16]. The MM-CGI-SF improves feasibility but shows limited validation among cancer caregiver populations [15]. The PG-12 is advantageous as it can be applied across caregiver populations regardless of the underlying illness and serves as both a pre-death grief assessment and a screening tool for PGD risk [13,17]. The most recent revision, the PG-12-R, incorporates refinements to improve psychometric performance [18]. Validation studies conducted in Portugal [13], Italy [19], and Germany [20] have demonstrated that the instrument has high reliability, indicating its efficacy across many cultures. However, there is no validated Thai version of the PG-12-R, limiting its use in Thai clinical and research settings.

Ramathibodi Hospital was selected as the study site due to its well-established palliative care service and the availability of caregivers for terminally ill cancer patients, allowing for the feasible recruitment of participants appropriate for psychometric validation. This study aims to translate and validate the PG-12-R in the Thai language. This tool is designed to support caregivers by assessing anticipatory grief, identifying individuals at risk for PGD, and facilitating the development of culturally relevant interventions tailored to the Thai context.

## Materials and methods

### Study design

This study employed a quantitative cross-sectional design consisting of two phases: the translation phase and the validation phase of the Prolonged Grief Disorder Questionnaire (PG-12-R) for use among caregivers of terminal cancer patients in Thailand.

### Translation phase

**Step 1: Forward translation.** The original PG-12-R was translated into Thai by two independent bilingual translators who are fluent in both English and Thai, understand the cultural context of grief expression in Thailand, and one of whom has expertise in medical terminology.
**Step 2: Reconciliation meeting.** To address and resolve any issues from the forward translation stage, a committee comprising bilingual medical professionals with expertise in palliative care and bereavement, as well as a bilingual specialist in Thai and English, was formed. This committee ensured the translation accurately conveyed the original instrument’s meaning while maintaining cultural relevance.
**Step 3: Back translation.** Blind to the original PG-12-R, a bilingual palliative care specialist and a bilingual specialist in both Thai and English translated the reconciled Thai version back into English. The reconciled back-translations are provided in (S1 Appendix).
**Step 4: Expert review.** The back-translated and reconciled Thai versions were presented to a group of experts, including linguists familiar with the Thai language and culture, as well as grief counselors acquainted with the PG-12-R and anticipatory grief assessment. Experts evaluated the translated tool for conceptual, experiential, idiomatic, and semantic equivalency.
**Step 5: Pilot testing.** A group of 30 Thai caregivers [21] of patients with terminal cancer in the palliative care service at Ramathibodi Hospital participated in a pilot study to assess the readability, flow, and clarity of the translated PG-12-R. Self-administered questionnaires included the reconciled Thai version of the PG-12-R, a brief sociodemographic questionnaire, and sections for suggestions and feedback. The translation was adjusted based on their suggestions.
**Step 6: Finalization.** Feedback from the expert review and pilot testing was used to improve the translated PG-12-R. Each committee member reviewed and approved the final version before the validation study. The final Thai version of the PG-12-R is available in (S2 Appendix).

### Validation phase

**Face validity.** To evaluate the clarity, comprehensiveness, and cultural appropriateness, 30 participants were interviewed during the pilot test. Participant feedback was subsequently incorporated to refine the wording and ensure the instrument’s appropriateness for the target population.
**Content validity.** The content validity index (CVI) was employed to assess content validity using a panel of experts: a psychologist, a psychiatrist, a family physician, and two palliative care specialists familiar with anticipatory grief assessment. They independently rated each PG-12-R item on a scale (1–4) based on its relevance and clarity. The comments from the panel of experts were taken into consideration when adjusting the instrument.
**Construct validity.** Construct validity was examined through structural and convergent validity. Structural validity was evaluated using exploratory factor analysis (EFA) to verify the underlying factor structure of the Thai PG-12-R within the Thai cultural context. Consistent with the original validation study of the PG-13-R, which established its unidimensionality through exploratory methods [22], EFA was employed here to ensure that the scale’s items remain conceptually and statistically cohesive in the Thai caregiver population. Convergent validity was assessed by examining the associations between the Thai PG-12-R and established measures of related psychological constructs, including anxiety (GAD-7), depression (PHQ-9), and caregiver burden (ZBI).
**Internal consistency.** Cronbach’s alpha coefficient was calculated to assess internal consistency.
**Test-retest reliability.** Test-retest was used to evaluate the stability of the PG-12-R scores over time, indicating whether the instrument yielded consistent results when administered twice within a reasonable time frame. Test-retest reliability was assessed using an intra-class correlation coefficient (ICC).

Criterion-related validity was not assessed in this study due to the lack of a formal diagnostic consensus or a gold standard assessment tool for anticipatory grief. Additionally, predictive validity for post-bereavement outcomes was not evaluated, as the study employed a cross-sectional design rather than a longitudinal approach.

The finalized Thai PG-12-R was administered to a larger sample of Thai caregivers of terminal cancer patients in the palliative care service at Ramathibodi Hospital. The sample size determination for Cronbach’s alpha and ICC resulted in a sample size of 165 participants, which was the largest. This estimation considered the ICC coefficient for hypothesis testing, with a power of 80%, a minimum acceptable reliability of 0.7, an expected reliability of 0.8, a significant level of 0.05, and an expected 10% dropout rate [23–26].

### Participants

Participants were Thai caregivers of terminally ill cancer patients receiving palliative care at Ramathibodi Hospital, Bangkok. The inclusion criteria were: 1) being a primary caregiver for a terminal cancer (end-stage cancer) patient, including family members, friends, and others with unpaid relationships with the patient, 2) being 18 years of age or older, and 3) possessing sufficient language proficiency to complete the questionnaires. Exclusion criteria included participants with missing data or those who were unable to be contacted for the test-retest analysis. The participants were recruited from October 8, 2024, to June 4, 2025.

### Data collection

Data were collected from caregivers who met the inclusion criteria during their visits to the palliative care service at Ramathibodi Hospital. A consecutive recruitment approach was employed, in which all eligible caregivers were invited to participate until the target sample size of 165 participants was achieved. After providing a thorough explanation of the study procedures, written informed consent was obtained before the data collection. Data were collected using self-administered questionnaires comprising the finalized Thai version of the PG-12-R, a brief sociodemographic questionnaire, the Thai version of the Generalized Anxiety Disorder-7 (GAD-7), the Patient Health Questionnaire-9 (PHQ-9), and the Short Form Zarit Burden Interview (ZBI).

To assess test–retest reliability, participants completed the Thai PG-12-R on two occasions, separated by a two-week interval. The second time, researchers contacted participants or used the Line application to schedule appointments for data collection. All interviews were conducted in a private setting to maintain confidentiality and minimize potential distractions.

### Study instrument

**The sociodemographic questionnaire** consisted of eight items, including age, gender, marital status, education level, relationship to the patient, whether the participant received care assistance (from a paid caregiver or another relative), care duration, and perceived intimacy with the patient (high, moderate, or low).

**The Prolonged Grief Disorder Questionnaire (PG-12-R)** is an adaptation of the PG-13-R, originally developed to assess Prolonged Grief Disorder (PGD) based on the DSM-5-TR criteria [18,22,27]. It consists of 13 items: 10 are rated on a five-point Likert scale, two are yes/no questions, and one is open-ended about the duration of the patient’s illness. In the pre-loss version, items were adapted to focus on illness-related experiences rather than bereavement. Total scores indicate the severity of pre-loss grief.

**The Thai validated Patient Health Questionnaire-9 (PHQ-9)** (Cronbach’s alpha = 0.79) [28] was used to assess depressive symptoms. With 10 items on a four-point Likert scale, higher scores indicate more severe depressive symptoms.

**The Thai validated Generalized Anxiety Disorder-7 (GAD-7)** (Cronbach’s alpha = 0.92) [29], with eight items using a four-point Likert scale, was employed to measure anxiety symptoms; higher scores indicate more anxiety.

**The Thai validated Zarit Burden Interview (ZBI)** (Cronbach’s alpha = 0.88) [30] was administered to assess caregiver burden. With 12 items on a five-point Likert scale, higher scores reflect a greater caregiver burden.

### Statistical analysis

Statistical analyses were performed using Stata Statistical Software, version 18.0 (StataCorp LLC, College Station, TX, USA), with a significance threshold set at α=0.05.

For the descriptive data, continuous data were described using mean and standard deviation (SD), or median and range if the data distribution was not normal. Meanwhile, categorical data were described using percentages and frequencies. The normality of continuous variables was assessed using the Shapiro-Wilk test.

To examine differences in demographic characteristics between participants with low and high scores (stratified by the median PG-12-R score), inferential statistics were applied. Continuous variables were compared using the independent *t*-test or the Mann-Whitney U test, depending on the data distribution. Categorical variables were compared using the Chi-square test ($\chi^{2}$) or Fisher’s exact test, as appropriate.

Construct validity was examined through structural and convergent validity. For structural validity, exploratory factor analysis (EFA) was conducted on the severity items using the iterated principal factor (IPF) method [31]. The inclusion item (Q1), the duration of the illness (Q2), and the functional impairment item (Q13) were excluded from the factor analysis as they serve distinct clinical purposes. The suitability of the data for factor analysis was assessed using the Kaiser-Meyer-Olkin (KMO) measure and the likelihood ratio test for independence. Factor loadings were inspected to verify the unidimensionality of the scale, and items with standardized factor loadings < 0.40 were considered for removal to ensure construct integrity [31,32]. All subsequent psychometric analyses were performed using the items retained in the final model.

Convergent validity was evaluated using Spearman’s correlation coefficients (ρ) and simple linear regression coefficients [33,34]. Spearman’s ρ was used to assess the strength and direction of the monotonic relationship for non-normally distributed data. Values between 0.30 and 0.50 indicated a fair correlation, 0.50 to 0.70 a moderate correlation, 0.70 to 0.90 a high correlation, and greater than 0.90 a very strong correlation [33]. Simultaneously, simple linear regression coefficients were calculated to quantify the magnitude of the effect, representing the estimated change in the PG-12-R score for every one-unit increase in the comparator measure [34].

A scale-level CVI (S-CVI) and item-level CVI (I-CVI) were calculated to assess the content validity. A calculated score of 0.80 or higher indicates good content validity, while a score of 0.90 or higher indicates excellent content validity [35,36].

Cronbach’s alpha coefficient was calculated to assess internal consistency. Reliability was interpreted using the following thresholds: values 0.70 or higher were considered acceptable, 0.80 or higher were considered good, and 0.90 or higher were considered excellent [37].

Regarding test-retest reliability, 165 participants [24,25] were asked to complete the translated PG-12-R twice, with a 2-week interval between administrations. Test-retest reliability was assessed using an intra-class correlation coefficient (ICC), along with its 95% confidence intervals (95% CI), which was calculated using Stata Statistical Software version 18.0 (Stata Corporation, College Station, TX), based on a single-rating, absolute-agreement, two-way mixed-effects model, ranging from 0 to 1. ICC values between 0.5 and 0.75 indicate moderate reliability, values between 0.75 and 0.9 indicate good reliability, and values greater than 0.9 indicate excellent reliability [38]. All underlying data for the face validity, content validity, and validation phases are provided in (S1 Dataset).

### Ethical considerations

The study was approved by the Human Research Ethics Committee, Faculty of Medicine Ramathibodi Hospital, Mahidol University (COA No. MURA2024/421, approved June 20, 2024), in line with the Declaration of Helsinki. The Thai version of PG-12-R was translated and published with permission from the original authors under the Creative Commons Attribution (CC BY) license.

## Results

### Participant demographic characteristics

During the recruitment period, 166 eligible caregivers were approached, and 165 agreed to participate, providing written informed consent, resulting in a participation rate of 99.4%. In total, 165 Thai caregivers of terminal cancer patients receiving palliative care at Ramathibodi Hospital were included in the study.

Following the exploratory factor analysis, the total score for the Thai PG-12-R was calculated using the 9 items retained in the final model (excluding Item Q3). Participants were then stratified into two groups based on the median of this modified total score (Median = 17): the Low Score group (*n* = 90, score ≤ 17) and the High Score group (*n* = 75, score > 17). Demographic characteristics of the total sample and the comparison between the two groups are summarized in Table 1.

**Table 1:** Participant characteristics.

Variables		Total Sample	Low Score	High Score	*p*-value
		(*n* = 165)	(*n* = 90)	(*n* = 75)	
**Gender, *n* (%)**	Male	48 (29.1)	27 (30.0)	21 (28.0)	0.778^a^
	Female	117 (70.9)	63 (70.0)	54 (72.0)	
**Age, years, mean (SD)**		49.2 (13.6)	50.2 (13.0)	48.0 (14.3)	0.301^b^
**Marital status, *n* (%)**	Single	48 (29.1)	25 (27.8)	23 (30.7)	0.542^a^
	Married	109 (66.1)	59 (65.6)	50 (66.7)	
	Divorced / Separated	6 (3.6)	4 (4.4)	2 (2.7)	
	Widowed	2 (1.2)	2 (2.2)	0 (0.0)	
**Education, *n* (%)**	Elementary school	10 (6.1)	7 (7.8)	3 (4.0)	0.305^a^
	Middle school	10 (6.1)	7 (7.8)	3 (4.0)	
	High school/ Vocational certificate	19 (11.5)	8 (8.9)	11 (14.7)	
	Bachelor’s degree/ High vocational certificate	73 (44.2)	43 (47.8)	30 (40.0)	
	Postgraduate	53 (32.1)	25 (27.8)	28 (37.3)	
**Relationship, *n* (%)**	Spouse	32 (19.4)	21 (23.3)	11 (14.7)	0.298^a^
	Children	93 (56.4)	44 (48.9)	49 (65.3)	
	Grandchild	8 (4.9)	6 (6.7)	2 (2.7)	
	Sibling	14 (8.5)	7 (7.8)	7 (9.3)	
	Parent	3 (1.8)	2 (2.2)	1 (1.3)	
	Other relatives	15 (9.1)	10 (11.1)	5 (6.7)	
**Care assistance, *n* (%)**	No	100 (60.6)	54 (60.0)	46 (61.3)	0.861^a^
	Yes	65 (39.4)	36 (40.0)	29 (38.7)	
**Care duration (months), median (IQR)**		24 (7, 48)	24 (7, 48)	24 (8, 60)	0.807^c^
**Perceived intimacy, *n* (%)**	High	151 (91.5)	81 (90.0)	70 (93.3)	0.566^a^
	Moderate	13 (7.9)	8 (8.9)	5 (6.7)	
	Low	1 (0.6)	1 (1.1)	0 (0.0)	

n = number, SD = standard deviation, IQR = interquartile range.

^a^Analyzed using Chi-square test.

^b^Analyzed using Independent *t*-test.

^c^Analyzed using Wilcoxon rank-sum (Mann-Whitney U) test.

Most participants were female (*n* = 117, 70.9%), while 48 participants were male (29.1%). The mean age of the participants was 49.2 years (SD = 13.6 years). Regarding marital status, most participants were married (*n* = 109, 66.1%), followed by single (*n* = 48, 29.1%), divorced or separated (*n* = 6, 3.6%), and widowed (*n* = 2, 1.2%). Regarding education level, the largest proportion of participants held a bachelor’s degree or high vocational certificate (*n* = 73, 44.2%), followed by postgraduate education (*n* = 53, 32.1%), high school or vocational certificate (*n* = 19, 11.5%), middle school (*n* = 10, 6.1%), and elementary school (*n* = 10, 6.1%). No participants reported having less than an elementary education. The relationship to the care recipient varied, with the majority being children (*n* = 93, 56.4%). Other relationships included spouses (*n* = 32, 19.4%), other relatives (*n* = 15, 9.1%), siblings (*n* = 14, 8.5%), grandchildren (*n* = 8, 4.9%), and parents (*n* = 3, 1.8%). No participants were friends or grandparents of the care recipients. Of the participants, 65 (39.4%) reported receiving care assistance, whereas 100 (60.6%) did not. The median duration of caregiving was 24 months (IQR, 7–48 months). Finally, the majority of participants reported a high level of intimacy with the care recipient (*n* = 151, 91.5%), followed by moderate intimacy (*n* = 13, 7.9%), and a low level of intimacy (*n* = 1, 0.6%).

Comparative analysis revealed no statistically significant differences between the Low Score and High Score groups regarding any demographic characteristics. Specifically, there were no significant differences in gender (*p* = 0.778), age (*p* = 0.301), marital status (*p* = 0.542), education level (*p* = 0.305), or relationship to the patient (*p* = 0.298). Furthermore, caregiving factors such as care assistance (*p* = 0.861), care duration (*p* = 0.807), and perceived intimacy (*p* = 0.566) were comparable between the two groups.

### Validity

#### Face validity.

Twenty-three participants (76.67%) reported that the translated items were clear and understandable. Furthermore, 27 participants (90.00%) felt that the items adequately addressed the anticipated grief experience in the Thai context. Finally, 28 participants (93.33%) affirmed the cultural relevance and sensitivity of the items to Thai expressions of grief.

#### Content validity.

The scale-level CVI (S-CVI) was calculated to be 0.92. Furthermore, the item-level CVI (I-CVI) was 1 for most items, except for Item 4, which received an I-CVI of 0.8. These values indicate excellent content validity, as presented in Table 2.

**Table 2:** Content validity index.

Item	Question	I-CVI	S-CVI
**Q1**	Are you caring for someone with an illness?	1	0.92
**Q2**	How many months has it been since the patient has been ill?	1	
**Q3**	Do you feel yourself longing or yearning for the patient to be healthy again?	1	
**Q4**	Do you have trouble doing the things you normally do because you are thinking so much about the patient’s illness?	0.8	
**Q5**	Do you feel confused about your role in life or a diminished sense of self (i.e., feeling that a part of yourself has died)?	1	
**Q6**	Have you had trouble accepting the patient’s illness?	1	
**Q7**	Do you avoid reminders that the patient is ill?	1	
**Q8**	Do you feel emotional pain (e.g., anger, bitterness, sorrow related to the patient’s illness)?	1	
**Q9**	Do you feel that you have trouble re-engaging in life (e.g., problems engaging with friends, pursuing interests, planning for the future)?	1	
**Q10**	Do you feel emotionally numb or detached from others?	1	
**Q11**	Do you feel that life is meaningless because of the patient’s illness?	1	
**Q12**	Do you feel alone or lonely since the patient’s illness?	1	
**Q13**	Have the symptoms above caused significant impairment in social, occupational, or other important areas of functioning?	1	

CVI = content validity index, I-CVI = item-level CVI, S-CVI = scale-level CVI.

### Construct validity

#### Exploratory Factor Analysis (EFA).

Exploratory Factor Analysis (EFA) was conducted to examine the underlying factor structure of the Thai PG-12-R using the iterated principal factor (IPF) method. The suitability of the data for factor analysis was confirmed by the Kaiser-Meyer-Olkin (KMO) measure of sampling adequacy, which was 0.83, indicating meritorious sampling adequacy. The likelihood ratio test for independence (testing the null hypothesis that variables are uncorrelated) was statistically significant ($\chi^{2}(36)=691.55,p<0.001$), supporting the factorability of the correlation matrix.

Initial analysis including all 10 severity items revealed that Item Q3 (“*Do you feel yourself longing or yearning for the patient to be healthy again?*”) exhibited a low factor loading (< 0.40). This suggests that in the context of anticipatory grief among Thai caregivers, the concept of “yearning” or “longing for health” may not consistently align with other grief severity symptoms. Consequently, Item Q3 was removed from the scale to ensure construct integrity.

The final EFA was performed on the remaining 9 items. The analysis yielded a distinct unidimensional structure, with a single factor having an eigenvalue of 4.27. This single factor accounted for 67.61% of the total variance. All 9 items loaded strongly onto the single factor, with standardized factor loadings ranging from 0.51 to 0.78.

#### Convergent validity.

Convergent validity was assessed by examining the correlations between the modified 9-item Thai PG-12-R total score and other established measures. As presented in Table 3, the Thai PG-12-R demonstrated moderate positive correlations with all comparator questionnaires (*p* < 0.001). The strongest correlation was observed with depression (PHQ-9: Spearman’s ρ=0.67), followed by caregiver burden (ZBI: ρ=0.60) and anxiety (GAD-7: ρ=0.59).

**Table 3:** Correlations between Thai PG-12-R and comparator questionnaires.

Questionnaires	Spearman’s ρ	*p*-value
PHQ-9	0.67	<0.001
GAD-7	0.59	<0.001
ZBI	0.60	<0.001

PHQ-9 = Patient Health Questionnaire-9, GAD-7 = Generalized Anxiety Disorder-7, ZBI = Zarit Burden Interview.

Simple linear regression analysis was further performed to quantify these associations (Table 4). The results indicated that the PHQ-9 (β=1.11, 95% CI: 0.94–1.28), GAD-7 (β=1.20, 95% CI: 0.96–1.44), and ZBI (β=0.54, 95% CI: 0.42–0.66) were all significant predictors of the modified Thai PG-12-R score.

**Table 4:** Simple linear regression analysis.

Questionnaires	Coefficient (β) [95% CI]	*p*-value
PHQ-9	1.11 [0.94-1.28]	<0.001
GAD-7	1.20 [0.96-1.44]	<0.001
ZBI	0.54 [0.42-0.66]	<0.001

PHQ-9 = Patient Health Questionnaire-9, GAD-7 = Generalized Anxiety Disorder-7, ZBI = Zarit Burden Interview, CI = confidence interval.

### Reliability

#### Internal consistency.

Following the exclusion of Item Q3 based on the EFA results, internal consistency was assessed for the remaining 9 severity items. The results indicated good internal consistency, with a Cronbach’s alpha value of 0.87.

#### Test-retest reliability.

Of the 165 participants who aimed to complete the translated PG-12-R twice with a two-week interval, 11 data sets were missing, resulting in a response rate of 93.33%. Consequently, 154 data sets were analyzed to assess test-retest reliability. The results in Table 5 show that individual items demonstrated moderate to good reliability, with ICC values ranging from 0.63 to 0.89, signifying a high degree of consistency across repeated measurements. Items Q2 (ICC = 0.89) and Q9 (ICC = 0.85) demonstrated good stability. Two items (Q6, Q11) had moderate reliability. The composite score for the modified 9-item scale demonstrated good reliability (ICC = 0.88), validating the overall stability of the instrument.

**Table 5:** Intra-class correlation coefficient of the test-retest reliability.

Item	Question	ICC [95% CI]
**Q1**	Are you caring for someone with an illness?	NA
**Q2**	How many months has it been since the patient has been ill?	0.89 [0.85-0.92]
**Q3**	Do you feel yourself longing or yearning for the patient to be healthy again?	0.74 [0.66-0.81]
**Q4**	Do you have trouble doing the things you normally do because you are thinking so much about the patient’s illness?	0.76 [0.68-0.82]
**Q5**	Do you feel confused about your role in life or a diminished sense of self (i.e., feeling that a part of yourself has died)?	0.72 [0.63-0.79]
**Q6**	Have you had trouble accepting the patient’s illness?	0.68 [0.58-0.75]
**Q7**	Do you avoid reminders that the patient is ill?	0.71 [0.63-0.78]
**Q8**	Do you feel emotional pain (e.g., anger, bitterness, sorrow related to the patient’s illness)?	0.71 [0.62-0.78]
**Q9**	Do you feel that you have trouble re-engaging in life (e.g., problems engaging with friends, pursuing interests, planning for the future)?	0.85 [0.80-0.89]
**Q10**	Do you feel emotionally numb or detached from others?	0.77 [0.70-0.83]
**Q11**	Do you feel that life is meaningless because of the patient’s illness?	0.63 [0.53-0.72]
**Q12**	Do you feel alone or lonely since the patient’s illness?	0.70 [0.61-0.77]
**Q13**	Have the symptoms above caused significant impairment in social, occupational, or other important areas of functioning?	NA
**Scale**		0.88 [0.84-0.92]

ICC = intraclass correlation coefficient, CI = confidence interval, NA = not applicable.

Items Q1 and Q13 were excluded from the internal consistency analysis because they serve separate objectives. Item Q1 functions as a screening question for inclusion criteria, while item Q13 assesses functional impairment to determine whether participants require further intervention, which is conceptually distinct from the symptom scale. Item Q3 was also excluded from the scale-level ICC calculation due to its poor structural validity.

## Discussion and conclusion

### Discussion

This study aimed to translate and validate the Prolonged Grief Disorder Questionnaire (PG-12-R) into Thai for use among caregivers of patients with terminal cancer. The findings indicate that the Thai PG-12-R is a reliable and valid instrument for assessing anticipatory grief in this population.

In terms of structural validity, the exploratory factor analysis (EFA) initially revealed that Item Q3 (“*Do you feel yourself longing or yearning for the patient to be healthy again?*”) exhibited a low factor loading (< 0.40). Consequently, this item was removed to optimize the unidimensional structure of the scale. This exclusion warrants discussion regarding both cultural nuances and response patterns. While “yearning” is a core symptom in Western models of prolonged grief, the Thai phrasing for Item Q3—translated as a desire for the patient to return to good health—may be perceived by Thai caregivers as a universal, instinctive wish or a form of benevolent prayer rather than a pathological symptom of grief. In the Thai cultural context, wishing for a loved one’s recovery is a natural expression of care and filial duty; thus, most participants likely endorsed this item at the highest level regardless of their actual grief severity. This resulted in a ceiling effect and limited variance, leading to the item’s weak correlation with other severity items. Furthermore, influenced by Buddhist philosophy, Thai caregivers often distinguish between the innate wish for health and the spiritual task of acceptance. They may experience intense emotional suffering while concurrently employing religious-based acceptance of impermanence (Anicca) as a way to endure the situation. This suggests that in the Thai context, spiritual acceptance of the patient’s condition does not necessarily diminish the instinctive desire for their recovery. Consequently, Item Q3 failed to differentiate levels of anticipatory grief in this population. After modification, the 9-item final model demonstrated a distinct unidimensional structure with high variance explained (67.61%), confirming that the remaining items cohesively measure the construct of anticipatory grief in the Thai context.

The Thai PG-12-R demonstrated good internal consistency (Cronbach’s alpha = 0.87). This finding is consistent with previous validations of the PG-12 and PG-12-R in other cultural contexts, such as the Portuguese version [13], the Italian version [19], and the recent German version [20], which reported an α of 0.9. The test–retest reliability, assessed over a two-week interval, showed an overall ICC of 0.88, indicating good temporal stability [38].

Face validity was supported by the majority of participants in the pilot study, who affirmed that the items were clear, culturally appropriate, and comprehensive in capturing anticipatory grief. Excellent content validity was indicated by the content validity index (S-CVI = 0.92; I-CVI ≥ 0.80 for all items) [35]. The Thai PG-12-R showed moderate correlations with established measures of anxiety (GAD-7, ρ = 0.59), depression (PHQ-9, ρ = 0.67), and caregiver burden (ZBI, ρ = 0.60), confirming construct validity. The simple linear regression analysis further elucidated the magnitude of these relationships; specifically, increases in anxiety and depression scores were associated with a more substantial rise in PG-12-R scores (coefficients > 1.0) compared to caregiver burden (coefficient = 0.54). This finding suggests that anticipatory grief in this population may be more closely tied to emotional distress than to the functional burden of caregiving. These results are consistent with earlier studies showing a strong correlation between emotional distress, caregiving stress, and anticipatory grief [5–7,13,20].

This study’s psychometric properties correspond with the Portuguese PG-12 validation [13], which also found strong correlations with related psychological constructs and high internal consistency. Additionally, the outcomes closely resemble the German PG-12-R validation [20], indicating the instrument’s adaptability to various linguistic and cultural contexts. These parallels support the PG-12-R’s cross-cultural suitability as a tool for evaluating pre-loss grief in patients’ caregivers.

### Implications for clinical practice

The validated Thai PG-12-R is a simple and culturally appropriate screening tool that may assist in identifying caregivers at high risk for anticipatory grief. Early detection may facilitate the prevention of prolonged grief disorder by allowing for targeted interventions and timely psychological support [8,9]. In alignment with global recommendations for holistic care that consider the needs of both patients and caregivers, these findings support the inclusion of the Thai PG-12-R in routine palliative care evaluations [2,7,13,20].

### Strengths and limitations

The translation and adaptation process, which produced both linguistic accuracy and cultural relevance while adhering to cross-cultural guidelines [21,39], is a key strength of the study. Additionally, the study assessed validity using various metrics, enhancing the psychometric evaluation.

However, several limitations should be noted. First, the majority of participants had a high level of education, including a bachelor’s degree, a high vocational certificate, and a postgraduate degree. The characteristics may limit its generalizability to caregivers in other settings, such as community hospitals, rural clinics, or home-based care. Second, Evaluation of predictive validity for long-term bereavement outcomes is not possible due to the cross-sectional design. Third, criterion-related validity (diagnostic accuracy) was not assessed. Unlike Prolonged Grief Disorder, anticipatory grief is not currently recognized as a formal diagnostic category in standard nosologies (e.g., DSM-5-TR or ICD-11); consequently, there is no established “gold standard” to distinguish clinical cases. At present, the Thai PG-12-R is best utilized as a continuous measure of symptom severity. However, further research is warranted to establish a clinical cut-off score, which would facilitate its use as a screening tool in palliative care settings. Finally, while test–retest reliability was acceptable, slight item-level variability suggests that certain grief-related symptoms may fluctuate in short timeframes.

### Future research

Future research should focus on longitudinal studies to determine the predictive validity of the Thai PG-12-R for post-bereavement prolonged grief disorder. Determining an optimal cut-off score for the Thai population would also enhance its clinical utility. Furthermore, multicenter studies involving various parts of Thailand would enhance generalizability. Incorporating the PG-12-R into long-term palliative care programs could help evaluate the effectiveness of treatments intended to lessen the severity of caregiver grief and distress.

### Conclusion

The PG-12-R was successfully translated into Thai, and the validation process demonstrated excellent content validity, good internal consistency and test-retest reliability, and satisfactory construct validity. Although one item was removed to optimize the model fit, the modified 9-item version serves as a robust tool for assessing grief severity. In clinical and research settings, it can help identify caregivers who are at risk of high anticipatory grief, allowing for timely and focused psychosocial support. By expanding knowledge and understanding of anticipatory grief in the Thai context, this tool has the potential to improve the quality of comprehensive palliative care in Thailand and the well-being of caregivers.

## Supporting information

S1 Appendix English Back-Translations of the Thai PG-12-R. (PDF)

S2 Appendix Thai PG-12-R. (PDF)

S1 Dataset Minimal underlying data set. This file contains the codebook, expert ratings for content validity, participant feedback for face validity, and raw data for the validation and test-retest phases.
(XLSX)
