# Supplementary material for: Cross-cultural adaptation and psychometric properties study of Prolonged Grief Disorder Questionnaire (PG-12-R) for caregivers of terminal cancer patients, Thai version
Source: PLoS One. 2026 Jul 15;21(7):e0343917. doi: 10.1371/journal.pone.0343917 (PMC13372141; doi:10.1371/journal.pone.0343917)
Supplement: S1 Appendix — (PDF) [file pone.0343917.s001.pdf]

PG-12 (Revised Edition)

1. Are you currently taking care of someone who is ill? ☐Yes ☐No

2. How long has the patient been ill? ....

For each question below, please indicate how you feel at the moment. Circle the number in the column on the right that corresponds to your answer.

	Not at all	Slightly	Moderately	Quite a lot	Extremely
3. Do you feel a longing or desire for the patient to return to their previous healthy state?	1	2	3	4	5
4. Do you have trouble doing things you used to do regularly because you are preoccupied with the patient's illness?	1	2	3	4	5
5. Do you feel confused about your role in life or feel like you've lost part of your identity (e.g., feeling like a part of you is missing)?	1	2	3	4	5
6. Do you have difficulty accepting the patient's illness?	1	2	3	4	5
7. Do you avoid reminders that the patient is ill?	1	2	3	4	5
8. Do you feel emotional pain (e.g., anger, bitterness, or sadness related to the patient's illness)?	1	2	3	4	5
9. Do you find it difficult to return to normal life activities such as meeting friends, pursuing your interests, or planning for the future?	1	2	3	4	5
10. Do you feel indifferent or want to distance yourself from others?	1	2	3	4	5
11. Do you feel life is meaningless because of the patient's illness?	1	2	3	4	5
12. Do you feel lonely or isolated since the patient became ill?	1	2	3	4	5

13. Have the above-mentioned symptoms significantly impacted your ability to socialize, work, or engage in other important activities? ☐Yes ☐No

PG-12 revised

1. Are you caring for someone who is sick? ☐Yes ☐No

2. How many months has the patient been sick for? ....

For each item below, please state how you are feeling right now. Please circle the number in the right column to indicate your answer.

	Not at all	A little	Somewhat	Somewhat a lot	A lot
3. Do you feel that you have longings or yearnings for the patient to return to good health again?	1	2	3	4	5
4. Do you have problems performing usual tasks because you're thinking a lot about the patient's illness?	1	2	3	4	5
5. Do you feel confused about your role in life or the loss of your identity (for example, feeling like you've lost a part of yourself)?	1	2	3	4	5
6. Do you have difficulty accepting the patient's illness?	1	2	3	4	5
7. Do you avoid things that remind you the patient is sick?	1	2	3	4	5
8. Do you feel emotional pain (for example angry, bitter or sad about the patient's illness)?	1	2	3	4	5
9. Do you feel you have difficulty in returning to your life, for example meeting friends, doing things that interest you or planning for the future?	1	2	3	4	5
10. Do you feel numb or detached from other people?	1	2	3	4	5
11. Do you feel life is meaningless because of the patient's illness?	1	2	3	4	5
12. Do you feel isolated or lonely since the patient became unwell?	1	2	3	4	5

13. Have the symptoms above caused significant problems in socializing, working or doing other important activities? ☐Yes ☐No
