# Supplementary material for: Cross-cultural adaptation and psychometric properties study of Prolonged Grief Disorder Questionnaire (PG-12-R) for caregivers of terminal cancer patients, Thai version
Source: PLoS One. 2026 Jul 15;21(7):e0343917. doi: 10.1371/journal.pone.0343917 (PMC13372141; doi:10.1371/journal.pone.0343917)
Supplement: S2 Appendix — (PDF) [file pone.0343917.s002.pdf]

## PG-12-ฉบับปรับปรุง

1. ท่านกำลังดูแลคนที่กำลังป่วยอยู่หรือไม่ ❑ ใช่ ❑ ไม่ใช่

2. ผู้ป่วยเจ็บป่วยมานานกี่เดือนแล้ว ......................................

สำหรับแต่ละข้อด้านล่าง โปรดระบุว่าท่านรู้สึกอย่างไรในขณะนี้ กรุณาวงกลมตัวเลขในคอลัมน์ด้านขวาเพื่อระบุคำตอบของท่าน

| | ไม่เลย | เล็กน้อย | ปานกลาง | ค่อนข้างมาก | มากที่สุด |
|---|---|---|---|---|---|
| 3. ท่านรู้สึกว่าตนเองปรารถนาให้ผู้ป่วยกลับมามีสุขภาพดีเหมือนเดิม | 1 | 2 | 3 | 4 | 5 |
| 4. ท่านมีปัญหาในการทำสิ่งที่เคยทำเป็นประจำ เพราะท่านกำลังคิดถึงแต่เรื่องความเจ็บป่วยของผู้ป่วย | 1 | 2 | 3 | 4 | 5 |
| 5. ท่านรู้สึกสับสนเกี่ยวกับบทบาทในชีวิตหรือรู้สึกสูญเสียตัวตนไป (เช่น รู้สึกว่าส่วนหนึ่งของท่านได้ขาดหายไป) | 1 | 2 | 3 | 4 | 5 |
| 6. ท่านมีปัญหาในการยอมรับความเจ็บป่วยของผู้ป่วย | 1 | 2 | 3 | 4 | 5 |
| 7. ท่านหลีกเลี่ยงสิ่งที่ย้ำเตือนว่าผู้ป่วยกำลังป่วยอยู่ | 1 | 2 | 3 | 4 | 5 |
| 8. ท่านรู้สึกเจ็บปวดทางจิตใจ (เช่น โกรธ ขมขื่น หรือโศกเศร้าเกี่ยวกับการเจ็บป่วยของผู้ป่วย) | 1 | 2 | 3 | 4 | 5 |
| 9. ท่านรู้สึกว่าท่านมีปัญหาในการใช้ชีวิต เช่น การพบปะเพื่อนฝูง การทำสิ่งที่ตนเองสนใจ หรือการวางแผนสำหรับอนาคต | 1 | 2 | 3 | 4 | 5 |
| 10. ท่านรู้สึกเฉยชาหรือต้องการตีตัวออกห่างจากผู้อื่น | 1 | 2 | 3 | 4 | 5 |
| 11. ท่านรู้สึกว่าชีวิตไร้ความหมายเพราะความเจ็บป่วยของผู้ป่วย | 1 | 2 | 3 | 4 | 5 |
| 12. ท่านรู้สึกโดดเดี่ยวหรือเหงาตั้งแต่ผู้ป่วยเริ่มเจ็บป่วย | 1 | 2 | 3 | 4 | 5 |

13. อาการดังกล่าวข้างต้นส่งผลให้ท่านมีปัญหาอย่างมากในการเข้าสังคม การทำงาน หรือการทำกิจกรรมอื่น ๆ ที่สำคัญ ❑ ใช่ ❑ ไม่ใช่

Source: **Prigerson, H. G.,** Viola, M., Brewin, C. R., Cox, C., Ouyang, D., Rogers, M., Pan, C. X., Rabin, S., Xu, J., Vaughan, S., Gordon-Elliot, J. S., Berlin, D., Lief, L., & Lichtenthal, W. G. (2019). Enhancing & Mobilizing the POtential for Wellness & Emotional Resilience (EMPOWER) among Surrogate Decision-Makers of ICU Patients: Study Protocol for a Randomized Controlled Trial. *Trials*, **20**, Article 408. https://doi.org/10.1186/s13063-019-3515-0
Thai translation by Arthit Chaithanasarn, et al., Faculty of Medicine Ramathibodi Hospital, Mahidol University, 2025.
Reproduced with permission granted by Dr. Holly G. Prigerson.
