# Supplementary material for: Cross-cultural adaptation and psychometric properties study of Prolonged Grief Disorder Questionnaire (PG-12-R) for caregivers of terminal cancer patients, Thai version
Source: PLoS One. 2026 Jul 15;21(7):e0343917. doi: 10.1371/journal.pone.0343917 (PMC13372141; doi:10.1371/journal.pone.0343917)
Supplement: Attachment — Submitted filename: Response to Reviewers.pdf [file pone.0343917.s004.pdf]

Date: December 11, 2025

To: The Editor-in-Chief **Journal:** PLOS ONE

Re: Response to reviewers for manuscript

Title: Cross-cultural adaptation and psychometric properties study of Prolonged Grief Disorder Questionnaire (PG-12-R) for caregivers of terminal cancer patients, Thai version

Dear Dr. Ibrahim,

Thank you for your email regarding our manuscript. We are grateful for the opportunity to revise our work and for the constructive comments provided by you and the reviewers. We have carefully reviewed the manuscript and have made significant revisions to address the points raised, which we believe have improved the quality and rigor of our study.

As you requested regarding the Financial Disclosure, we confirm the following statement:

"The funders had no role in study design, data collection and analysis, decision to publish, or preparation of the manuscript."

Please find below a point-by-point response detailing how each comment has been addressed in the revised manuscript.

Point-by-Point Response to Editor Comments

Comment 1: Although factor analysis is not mandatory for a translation study, the authors should explicitly justify the absence of both exploratory factor analysis (EFA) and confirmatory factor analysis (CFA).

Response: Thank you for this suggestion. We have added a clear justification in the Materials and methods section (Validation phase: Construct validity). We explained that we did not perform EFA or CFA because the primary aim of this study was to establish the cross-cultural equivalence and psychometric properties (specifically, reliability and convergent validity) of the translated instrument. Furthermore, since the PG-12-R is a direct adaptation of the PG-13-R, its underlying factor structure has already been established in the original language.

Comment 2: The manuscript should also clearly explain why items Q1, Q2, and Q13 were excluded from the reliability analysis.

Response: We have included a specific explanation in the Results section (Reliability: Test-retest reliability). We clarified that items Q1 (screening) and Q13 (functional impairment) serve separate objectives and are conceptually distinct from the symptom severity scale. Therefore, they were excluded from the internal consistency analysis.

Comment 3: The sampling approach requires clearer justification: the authors should elaborate on why purposive sampling was chosen, describe the number of caregivers approached versus those enrolled, and discuss potential limitations related to representativeness and external validity.

Response: We have revised this section to be more accurate and transparent regarding our methodology:

Sampling Method: In the Data collection section, we have updated the text to reflect that a consecutive recruitment approach was employed (instead of purposive sampling) to reduce selection bias. We invited all eligible caregivers visiting the palliative care service until the target sample size was reached.

Enrollment Numbers: We have reported that 166 eligible caregivers were approached, and 165 agreed to participate, resulting in a participation rate of 99.4%.

Comment 4: Statistical reporting should also be refined, particularly correcting the inconsistent ICC confidence interval and ensuring a consistent interpretation of Cronbach's alpha thresholds.

Response: We have re-verified our statistical analysis and made the following corrections:

ICC Values: We have updated the results to reflect the correct calculated values. The overall scale ICC is 0.87 [95% CI: 0.83–0.90], and Item Q2 shows an ICC of 0.89. These updates are reflected in the Abstract, Results, and Table 2.

Cronbach's Alpha Thresholds: In the Statistical analysis section, we explicitly defined the interpretation thresholds (≥ 0.70 acceptable, ≥ 0.80 good, ≥ 0.90 excellent). In the Results, we consistently interpreted the alpha of 0.85 as indicating "good" internal consistency.

Comment 5: The rationale for employing both regression coefficients and correlation coefficients when assessing convergent validity should be clarified to avoid confusion.

Response: We have clarified the rationale in the Statistical analysis section. We explain that Spearman's ρ was used to assess the strength and direction of the monotonic relationship for non-normally distributed data. Simultaneously, regression coefficients were calculated to quantify the magnitude of the effect, representing the estimated change in the PG-12-R score for every one-unit increase in the comparator measure. We also expanded the Discussion section to highlight how the regression results provide insight into the stronger impact of emotional distress compared to caregiver burden.

Comment 6: Minor improvements in table formatting, English language flow, and alignment with PLOS ONE formatting guidelines would further enhance readability.

Response: We have thoroughly reviewed and formatted the manuscript according to PLOS ONE guidelines:

Tables: We have updated Tables 1–4 to align with the template structure, ensuring consistent column alignment.

Statistical Symbols: We have ensured that statistical symbols such as the p-value are correctly italicized (e.g., $p < 0.001$) throughout the text and tables.

Language: We have polished the English language throughout the manuscript to improve flow and correct minor grammatical errors.

Comment 7: Additionally, the introduction and discussion should be strengthened by improving narrative coherence and moderating clinical claims to align more closely with PLOS ONE standards.

Response: We have revised the Introduction and Discussion sections to improve narrative flow. We have also moderated our clinical claims, replacing definitive terms (e.g., "prove," "prevent") with more scientifically appropriate language (e.g., "indicate," "may facilitate," "assist in identifying").

Comment 8: Funding information.

Response: We confirm that all funding-related text has been removed from the manuscript body. The amended funding statement has been included in the Cover Letter as requested.

We believe these revisions fully address the concerns raised. We have uploaded a marked-up copy of the manuscript highlighting these changes, as well as a clean version.

Thank you again for your time and consideration.

Sincerely,

Arthit Chaithanasarn, M.D.

Department of Family Medicine, Faculty of Medicine Ramathibodi Hospital,
Mahidol University, Bangkok, Thailand
E-mail: arthit.cht@mahidol.ac.th
(On behalf of all co-authors)
