# Supplementary material for: Cross-cultural adaptation and psychometric properties study of Prolonged Grief Disorder Questionnaire (PG-12-R) for caregivers of terminal cancer patients, Thai version
Source: PLoS One. 2026 Jul 15;21(7):e0343917. doi: 10.1371/journal.pone.0343917 (PMC13372141; doi:10.1371/journal.pone.0343917)
Supplement: Attachment — Submitted filename: Response_to_Reviewers_auresp_2.pdf [file pone.0343917.s005.pdf]

Dear Editor and Reviewers,

We thank the editorial team and the reviewers for their constructive feedback and insightful suggestions on our manuscript. We have carefully addressed all the points raised to enhance the scientific rigor and clarity of our study. The revisions are highlighted in the 'Revised Manuscript with Track Changes'.

Responses to Reviewer #1 (Dr. Chavit Tunvirachaisakul)

1. REPORTING CONSTRUCT VALIDITY TESTING (EFA)

- **Reviewer Comment:** The manuscript does not report construct validity testing (e.g., factor analysis). This substantially weakens the overall psychometric quality of the study, particularly given that the number of items was modified. According to COSMIN guidelines, assessment of structural validity is essential in cross-cultural adaptation studies, and becomes even more critical when item reduction is performed.
- **Author Response:** We agree with the reviewer's concern regarding the necessity of structural validity. In this revised version, we have performed and reported **Exploratory Factor Analysis (EFA)** using the iterated principal factor (IPF) method.
- **Revision details:**
    - Initial EFA revealed that **Item Q3** had a low factor loading (< 0.40) and was subsequently removed to ensure construct integrity.
    - The final 9-item model demonstrated a clear **unidimensional structure** (Eigenvalue = 4.27, accounting for 67.61% of total variance).

2. CLINICAL (CRITERION-RELATED) VALIDITY

- **Reviewer Comment:** While content validity based on expert consensus is acceptable as an initial step, it is insufficient on its own. Evidence of clinical (criterion-related) validity is needed, ideally using diagnosis or structured clinical assessment by trained specialists as an external standard. This would allow evaluation of the accuracy of the Thai version and enable comparison with the original instrument.
- **Author Response:** We acknowledge this limitation. As anticipatory grief is currently not a formal diagnostic category in the DSM-5-TR or ICD-11, there is no established "gold standard" tool to provide a definitive clinical cut-off for case detection.
- **Revision details:** We have added a detailed explanation in the **Strengths and Limitations** section. We clarified that the Thai PG-12-R is best used as a continuous measure of symptom severity at this stage, and we have prioritized establishing a clinical cut-off score as a key objective for future research.

3. SEPARATING REGRESSION AND CORRELATION COEFFICIENTS

- **Reviewer Comment:** Regression coefficients and correlation coefficients are presented within the same table. This presentation is confusing, as these statistics address different analytical questions. Please clarify the analytic intent and separate these results into distinct tables or sections, with clear labeling.
- **Author Response:** We appreciate this suggestion for better clarity. We have now separated these statistics into distinct tables to address different analytical questions more clearly.

- **Revision details:**
    - **Table 3** now presents **Convergent Validity** through Spearman's correlation coefficients.
    - **Table 4** now presents the **Simple Linear Regression Analysis** to quantify the predictive effect of related constructs on grief scores.

4. STRATIFYING TABLE 1 BY GRIEF SCORES

- **Reviewer Comment:** Table 1 would be more informative if the sample were stratified into low-score and high-score groups, in addition to presenting the total sample. This would improve interpretability and clinical relevance.
- **Author Response:** Thank you for this clinical insight. This stratification provides a much clearer picture of the participant characteristics.
- **Revision details: Table 1** has been updated to include three columns: Total Sample, Low Score group, and High Score group (stratified by the median score of 17). Comparative analyses (p-values) between the groups have also been added.

Responses to Journal Requirements & Reference List

1. **Reference List**: We have thoroughly reviewed the reference list. We have removed "Available from" and URL links for entries that have a DOI, following PLOS ONE's preference for cleaner citations.
2. **New Citations**: We have added references for EFA methodology (Hair et al., 2010; Costello & Osborne, 2005) to support the added analysis.
3. **Table Order**: We have ensured all tables are cited in the text in chronological order:
    - Table 1: Participant characteristics (Stratified)
    - Table 2: Content Validity Index (CVI)
    - Table 3: Convergent Validity (Correlation)
    - Table 4: Simple Linear Regression
    - Table 5: Intraclass Correlation Coefficient (ICC)
4. **Structural Reorganization**: We have reorganized the Results section to present Validity findings (Face, Content, and Construct Validity) before Reliability (Internal Consistency and Test-Retest). This reordering aligns with the structure of the Methods section and reflects a logical progression of psychometric evaluation.
5. **Data Availability and Correction**:
    - Raw Data: In compliance with PLOS ONE's data policy, we have submitted the "S3_Dataset.xlsx" file containing all raw data from the Face Validity, Content Validity, Main Study, and Test-Retest phases.
    - Language: All data labels, categorical codes, and qualitative comments from experts and pilot participants have been translated into English to facilitate data re-use and transparency.
    - Data Correction (Care Assistance): During the preparation of the final dataset, we identified a coding error in the "Care assistance" variable within the demographic data, where "Yes" and "No" responses were inadvertently swapped. This error has been corrected in both the final dataset and the updated Table 1 in the manuscript. This correction did not affect the primary psychometric findings or the scale's structural integrity.

We hope that these revisions adequately address the reviewers' concerns and that the manuscript is now suitable for publication in PLOS ONE.

Sincerely,

Arthit Chaithanasarn, M.D.

Department of Family Medicine, Faculty of Medicine Ramathibodi Hospital,
Mahidol University, Bangkok, Thailand
E-mail: arthit.cht@mahidol.ac.th
(On behalf of all co-authors)
