# Supplementary material for: Cross-cultural adaptation and psychometric properties study of Prolonged Grief Disorder Questionnaire (PG-12-R) for caregivers of terminal cancer patients, Thai version
Source: PLoS One. 2026 Jul 15;21(7):e0343917. doi: 10.1371/journal.pone.0343917 (PMC13372141; doi:10.1371/journal.pone.0343917)
Supplement: Peer review history

# Editor report: Decision Letter 0

**DOI:** 10.1371/journal.pone.0343917.r001

**Authors:** Mohd Ismail Ibrahim

22 Nov 2025

PONE-D-25-55651

Cross-cultural adaptation and psychometric properties study of Prolonged Grief Disorder Questionnaire (PG-12-R) for caregivers of terminal cancer patients, Thai version

PLOS ONE

Dear Dr. Chaithanasarn,

Thank you for submitting your manuscript to PLOS ONE. After careful consideration, we feel that it has merit but does not fully meet PLOS ONE’s publication criteria as it currently stands. Therefore, we invite you to submit a revised version of the manuscript that addresses the points raised during the review process.

Please submit your revised manuscript by Jan 06 2026 11:59PM. If you will need more time than this to complete your revisions, please reply to this message or contact the journal office at plosone@plos.org. When you're ready to submit your revision, log on to https://www.editorialmanager.com/pone/ and select the 'Submissions Needing Revision' folder to locate your manuscript file.

Please include the following items when submitting your revised manuscript:

- A rebuttal letter that responds to each point raised by the academic editor and reviewer(s). You should upload this letter as a separate file labeled 'Response to Reviewers'.
- A marked-up copy of your manuscript that highlights changes made to the original version. You should upload this as a separate file labeled 'Revised Manuscript with Track Changes'.
- An unmarked version of your revised paper without tracked changes. You should upload this as a separate file labeled 'Manuscript'.

If you would like to make changes to your financial disclosure, please include your updated statement in your cover letter. Guidelines for resubmitting your figure files are available below the reviewer comments at the end of this letter.

If applicable, we recommend that you deposit your laboratory protocols in protocols.io to enhance the reproducibility of your results. Protocols.io assigns your protocol its own identifier (DOI) so that it can be cited independently in the future. For instructions see: https://journals.plos.org/plosone/s/submission-guidelines#loc-laboratory-protocols. Additionally, PLOS ONE offers an option for publishing peer-reviewed Lab Protocol articles, which describe protocols hosted on protocols.io. Read more information on sharing protocols at https://plos.org/protocols?utm_medium=editorial-email&utm_source=authorletters&utm_campaign=protocols.

We look forward to receiving your revised manuscript.

Kind regards,

Mohd Ismail Ibrahim, MCom.Med

Academic Editor

PLOS ONE

**Journal Requirements:**

When submitting your revision, we need you to address these additional requirements.

1. Please ensure that your manuscript meets PLOS ONE's style requirements, including those for file naming. The PLOS ONE style templates can be found at

https://journals.plos.org/plosone/s/file?id=wjVg/PLOSOne_formatting_sample_main_body.pdf and

https://journals.plos.org/plosone/s/file?id=ba62/PLOSOne_formatting_sample_title_authors_affiliations.pdf

2. Please update your submission to use the PLOS LaTeX template. The template and more information on our requirements for LaTeX submissions can be found at http://journals.plos.org/plosone/s/latex.

3. Thank you for stating the following financial disclosure:

“- Initials of the authors who received each award: AS

- Grant numbers awarded to each author: none

- The full name of each funder: Faculty of Medicine, Ramathibodi Hospital, Mahidol University

- URL of each funder website: https://www.rama.mahidol.ac.th/research/th/fund/faculty

- Did the sponsors or funders play any role in the study design, data collection and analysis, decision to publish, or preparation of the manuscript?: No”

Please state what role the funders took in the study.  If the funders had no role, please state: "The funders had no role in study design, data collection and analysis, decision to publish, or preparation of the manuscript."

If this statement is not correct you must amend it as needed.

Please include this amended Role of Funder statement in your cover letter; we will change the online submission form on your behalf.

4. Please note that funding information should not appear in any section or other areas of your manuscript. We will only publish funding information present in the Funding Statement section of the online submission form. Please remove any funding-related text from the manuscript.

5. If the reviewer comments include a recommendation to cite specific previously published works, please review and evaluate these publications to determine whether they are relevant and should be cited. There is no requirement to cite these works unless the editor has indicated otherwise.

**Additional Editor Comments:**

The manuscript is generally suitable for peer review; however, several methodological and reporting issues should be addressed beforehand to enhance rigor and avoid predictable reviewer concerns. Although factor analysis is not mandatory for a translation study, the authors should explicitly justify the absence of both exploratory factor analysis (EFA) and confirmatory factor analysis (CFA), as CFA is often expected to verify whether the translated scale retains the original instrument’s dimensional structure. The manuscript should also clearly explain why items Q1, Q2, and Q13 were excluded from the reliability analysis. Importantly, the sampling approach requires clearer justification: the authors should elaborate on why purposive sampling was chosen, describe the number of caregivers approached versus those enrolled, and discuss potential limitations related to representativeness and external validity. Statistical reporting should also be refined, particularly correcting the inconsistent ICC confidence interval and ensuring a consistent interpretation of Cronbach’s alpha thresholds. The rationale for employing both regression coefficients and correlation coefficients when assessing convergent validity should be clarified to avoid confusion. Minor improvements in table formatting, English language flow, and alignment with PLOS ONE formatting guidelines would further enhance readability. Additionally, the introduction and discussion should be strengthened by improving narrative coherence and moderating clinical claims to align more closely with PLOS ONE standards. Addressing these issues will substantially improve clarity, methodological robustness, and overall manuscript quality before it proceeds to peer review.

[Note: HTML markup is below. Please do not edit.]

[NOTE: If reviewer comments were submitted as an attachment file, they will be attached to this email and accessible via the submission site. Please log into your account, locate the manuscript record, and check for the action link "View Attachments". If this link does not appear, there are no attachment files.]

To ensure your figures meet our technical requirements, please review our figure guidelines: https://journals.plos.org/plosone/s/figures

You may also use PLOS’s free figure tool, NAAS, to help you prepare publication quality figures: https://journals.plos.org/plosone/s/figures#loc-tools-for-figure-preparation.

NAAS will assess whether your figures meet our technical requirements by comparing each figure against our figure specifications.

# Author comment: Author response to Decision Letter 1

**DOI:** 10.1371/journal.pone.0343917.r002

12 Dec 2025

Response to Reviewers for Manuscript: “Cross-cultural adaptation and psychometric properties study of Prolonged Grief Disorder Questionnaire (PG-12-R) for caregivers of terminal cancer patients, Thai version”

Dear Dr. Ibrahim and Reviewers,

Thank you for your email regarding our manuscript. We are grateful for the opportunity to revise our work and for the constructive comments provided by you and the reviewers. We have carefully reviewed the manuscript and have made significant revisions to address the points raised, which we believe have improved the quality and rigor of our study.

As you requested regarding the Financial Disclosure, we confirm the following statement:

"The funders had no role in study design, data collection and analysis, decision to publish, or preparation of the manuscript."

Please find below a point-by-point response detailing how each comment has been addressed in the revised manuscript.

________________________________________

Point-by-Point Response to Editor Comments

Comment 1: Although factor analysis is not mandatory for a translation study, the authors should explicitly justify the absence of both exploratory factor analysis (EFA) and confirmatory factor analysis (CFA).

Response: Thank you for this suggestion. We have added a clear justification in the Materials and methods section (Validation phase: Construct validity). We explained that we did not perform EFA or CFA because the primary aim of this study was to establish the cross-cultural equivalence and psychometric properties (specifically, reliability and convergent validity) of the translated instrument. Furthermore, since the PG-12-R is a direct adaptation of the PG-13-R, its underlying factor structure has already been established in the original language.

Comment 2: The manuscript should also clearly explain why items Q1, Q2, and Q13 were excluded from the reliability analysis.

Response: We have included a specific explanation in the Results section (Reliability: Test-retest reliability). We clarified that items Q1 (screening) and Q13 (functional impairment) serve separate objectives and are conceptually distinct from the symptom severity scale. Therefore, they were excluded from the internal consistency analysis.

Comment 3: The sampling approach requires clearer justification: the authors should elaborate on why purposive sampling was chosen, describe the number of caregivers approached versus those enrolled, and discuss potential limitations related to representativeness and external validity.

Response: We have revised this section to be more accurate and transparent regarding our methodology:

Sampling Method: In the Data collection section, we have updated the text to reflect that a consecutive recruitment approach was employed (instead of purposive sampling) to reduce selection bias. We invited all eligible caregivers visiting the palliative care service until the target sample size was reached.

Enrollment Numbers: We have reported that 166 eligible caregivers were approached, and 165 agreed to participate, resulting in a participation rate of 99.4%.

Comment 4: Statistical reporting should also be refined, particularly correcting the inconsistent ICC confidence interval and ensuring a consistent interpretation of Cronbach’s alpha thresholds.

Response: We have re-verified our statistical analysis and made the following corrections:

ICC Values: We have updated the results to reflect the correct calculated values. The overall scale ICC is 0.87 [95% CI: 0.83–0.90], and Item Q2 shows an ICC of 0.89. These updates are reflected in the Abstract, Results, and Table 2.

Cronbach’s Alpha Thresholds: In the Statistical analysis section, we explicitly defined the interpretation thresholds (≥ 0.70 acceptable, ≥ 0.80 good, ≥ 0.90 excellent). In the Results, we consistently interpreted the alpha of 0.85 as indicating "good" internal consistency.

Comment 5: The rationale for employing both regression coefficients and correlation coefficients when assessing convergent validity should be clarified to avoid confusion.

Response: We have clarified the rationale in the Statistical analysis section. We explain that Spearman’s ρ was used to assess the strength and direction of the monotonic relationship for non-normally distributed data. Simultaneously, regression coefficients were calculated to quantify the magnitude of the effect, representing the estimated change in the PG-12-R score for every one-unit increase in the comparator measure. We also expanded the Discussion section to highlight how the regression results provide insight into the stronger impact of emotional distress compared to caregiver burden.

Comment 6: Minor improvements in table formatting, English language flow, and alignment with PLOS ONE formatting guidelines would further enhance readability.

Response: We have thoroughly reviewed and formatted the manuscript according to PLOS ONE guidelines:

Tables: We have updated Tables 1–4 to align with the template structure, ensuring consistent column alignment.

Statistical Symbols: We have ensured that statistical symbols such as the p-value are correctly italicized (e.g., p < 0.001) throughout the text and tables.

Language: We have polished the English language throughout the manuscript to improve flow and correct minor grammatical errors.

Comment 7: Additionally, the introduction and discussion should be strengthened by improving narrative coherence and moderating clinical claims to align more closely with PLOS ONE standards.

Response: We have revised the Introduction and Discussion sections to improve narrative flow. We have also moderated our clinical claims, replacing definitive terms (e.g., "prove," "prevent") with more scientifically appropriate language (e.g., "indicate," "may facilitate," "assist in identifying").

Comment 8: Funding information.

Response: We confirm that all funding-related text has been removed from the manuscript body. The amended funding statement has been included in the Cover Letter as requested.

Attachment Submitted filename: Response to Reviewers.pdf

# Peer review: Decision Letter 1

**DOI:** 10.1371/journal.pone.0343917.r003

**Authors:** Mohd Ismail Ibrahim

6 Jan 2026

PONE-D-25-55651R1

Cross-cultural adaptation and psychometric properties study of Prolonged Grief Disorder Questionnaire (PG-12-R) for caregivers of terminal cancer patients, Thai version

PLOS One

Dear Dr. Chaithanasarn,

Thank you for submitting your manuscript to PLOS ONE. After careful consideration, we feel that it has merit but does not fully meet PLOS ONE’s publication criteria as it currently stands. Therefore, we invite you to submit a revised version of the manuscript that addresses the points raised during the review process.

Please submit your revised manuscript by Feb 20 2026 11:59PM. If you will need more time than this to complete your revisions, please reply to this message or contact the journal office at plosone@plos.org. When you're ready to submit your revision, log on to https://www.editorialmanager.com/pone/ and select the 'Submissions Needing Revision' folder to locate your manuscript file.

Please include the following items when submitting your revised manuscript:

- A letter that responds to each point raised by the academic editor and reviewer(s). You should upload this letter as a separate file labeled 'Response to Reviewers'.
- A marked-up copy of your manuscript that highlights changes made to the original version. You should upload this as a separate file labeled 'Revised Manuscript with Track Changes'.
- An unmarked version of your revised paper without tracked changes. You should upload this as a separate file labeled 'Manuscript'.

-->If you would like to make changes to your financial disclosure, please include your updated statement in your cover letter. Guidelines for resubmitting your figure files are available below the reviewer comments at the end of this letter.

If applicable, we recommend that you deposit your laboratory protocols in protocols.io to enhance the reproducibility of your results. Protocols.io assigns your protocol its own identifier (DOI) so that it can be cited independently in the future. For instructions see: https://journals.plos.org/plosone/s/submission-guidelines#loc-laboratory-protocols. Additionally, PLOS ONE offers an option for publishing peer-reviewed Lab Protocol articles, which describe protocols hosted on protocols.io. Read more information on sharing protocols at https://plos.org/protocols?utm_medium=editorial-email&utm_source=authorletters&utm_campaign=protocols.

We look forward to receiving your revised manuscript.

Kind regards,

Mohd Ismail Ibrahim, MCom.Med

Academic Editor

PLOS One

**Journal Requirements:**

1. If the reviewer comments include a recommendation to cite specific previously published works, please review and evaluate these publications to determine whether they are relevant and should be cited. There is no requirement to cite these works unless the editor has indicated otherwise.

2. Please review your reference list to ensure that it is complete and correct. If you have cited papers that have been retracted, please include the rationale for doing so in the manuscript text, or remove these references and replace them with relevant current references. Any changes to the reference list should be mentioned in the rebuttal letter that accompanies your revised manuscript. If you need to cite a retracted article, indicate the article’s retracted status in the References list and also include a citation and full reference for the retraction notice.

[Note: HTML markup is below. Please do not edit.]

Reviewers' comments:

Reviewer's Responses to Questions

**Comments to the Author**

1. If the authors have adequately addressed your comments raised in a previous round of review and you feel that this manuscript is now acceptable for publication, you may indicate that here to bypass the “Comments to the Author” section, enter your conflict of interest statement in the “Confidential to Editor” section, and submit your "Accept" recommendation.

Reviewer #1: All comments have been addressed

Reviewer #2: All comments have been addressed

**********

2. Is the manuscript technically sound, and do the data support the conclusions?

The manuscript must describe a technically sound piece of scientific research with data that supports the conclusions. Experiments must have been conducted rigorously, with appropriate controls, replication, and sample sizes. The conclusions must be drawn appropriately based on the data presented.

Reviewer #1: Partly

Reviewer #2: Yes

**********

3. Has the statistical analysis been performed appropriately and rigorously?

Reviewer #1: No

Reviewer #2: Yes

**********

4. Have the authors made all data underlying the findings in their manuscript fully available?

The PLOS Data policy requires authors to make all data underlying the findings described in their manuscript fully available without restriction, with rare exception (please refer to the Data Availability Statement in the manuscript PDF file). The data should be provided as part of the manuscript or its supporting information, or deposited to a public repository. For example, in addition to summary statistics, the data points behind means, medians and variance measures should be available. If there are restrictions on publicly sharing data—e.g. participant privacy or use of data from a third party—those must be specified.

Reviewer #1: Yes

Reviewer #2: Yes

**********

5. Is the manuscript presented in an intelligible fashion and written in standard English?

PLOS ONE does not copyedit accepted manuscripts, so the language in submitted articles must be clear, correct, and unambiguous. Any typographical or grammatical errors should be corrected at revision, so please note any specific errors here.

Reviewer #1: Yes

Reviewer #2: Yes

**********

6. Review Comments to the Author

Please use the space provided to explain your answers to the questions above. You may also include additional comments for the author, including concerns about dual publication, research ethics, or publication ethics. (Please upload your review as an attachment if it exceeds 20,000 characters)

Reviewer #1: Thank you for the opportunity to review your manuscript entitled “Cross-cultural adaptation and psychometric properties of the Prolonged Grief Disorder Questionnaire (PG-12-R) for caregivers of terminal cancer patients, Thai version.” The topic is clinically relevant and timely. However, I have some concerns regarding the validity assessment of the translated instrument that should be addressed to strengthen the scientific rigor of the study.

First, the manuscript does not report construct validity testing (e.g., factor analysis). This substantially weakens the overall psychometric quality of the study, particularly given that the number of items was modified. According to COSMIN guidelines (https://www.cosmin.nl/) , assessment of structural validity is essential in cross-cultural adaptation studies, and becomes even more critical when item reduction is performed.

Second, while content validity based on expert consensus is acceptable as an initial step, it is insufficient on its own. Evidence of clinical (criterion-related) validity is needed, ideally using diagnosis or structured clinical assessment by trained specialists as an external standard. This would allow evaluation of the accuracy of the Thai version and enable comparison with the original instrument. (please see https://he01.tci-thaijo.org/index.php/JPAT/article/view/260022)

Third, regression coefficients and correlation coefficients are presented within the same table. This presentation is confusing, as these statistics address different analytical questions. Please clarify the analytic intent and separate these results into distinct tables or sections, with clear labeling.

Finally, Table 1 would be more informative if the sample were stratified into low-score and high-score groups, in addition to presenting the total sample. This would improve interpretability and clinical relevance.

Reviewer #2: (No Response)

**********

7. PLOS authors have the option to publish the peer review history of their article (what does this mean?). If published, this will include your full peer review and any attached files.

If you choose “no”, your identity will remain anonymous but your review may still be made public.

**Do you want your identity to be public for this peer review?**  For information about this choice, including consent withdrawal, please see our Privacy Policy.

Reviewer #1: **Yes:**  Chavit Tunvirachaisakul, MD PhD

Reviewer #2: No

**********

[NOTE: If reviewer comments were submitted as an attachment file, they will be attached to this email and accessible via the submission site. Please log into your account, locate the manuscript record, and check for the action link "View Attachments". If this link does not appear, there are no attachment files.]

To ensure your figures meet our technical requirements, please review our figure guidelines: https://journals.plos.org/plosone/s/figures

You may also use PLOS’s free figure tool, NAAS, to help you prepare publication quality figures: https://journals.plos.org/plosone/s/figures#loc-tools-for-figure-preparation.

NAAS will assess whether your figures meet our technical requirements by comparing each figure against our figure specifications.

# Author comment: Author response to Decision Letter 2

**DOI:** 10.1371/journal.pone.0343917.r004

28 Jan 2026

Dear Editor and Reviewers,

We would like to express our sincere gratitude to the editorial team and the reviewers for their time and valuable feedback. We have addressed all comments to strengthen the scientific rigor of our manuscript. The following is a point-by-point response to the reviewers' concerns.

### Responses to Reviewer #1 (Dr. Chavit Tunvirachaisakul)

1. REPORTING CONSTRUCT VALIDITY TESTING (FACTOR ANALYSIS): We entirely agree that structural validity is crucial. In this revision, we have performed Exploratory Factor Analysis (EFA) using the Iterated Principal Factor (IPF) method to evaluate the internal structure of the Thai PG-12-R.

Revision details: The EFA results indicated that Item Q3 ("longing for the patient to be healthy again") had a factor loading below the 0.40 threshold. Consequently, we removed this item to achieve a robust unidimensional structure. This resulted in a 9-item scale with a high cumulative variance explained of 67.61%. We have detailed these statistical findings within the Results section (Construct Validity).

2. CLINICAL (CRITERION-RELATED) VALIDITY: We appreciate this important point. Currently, a "gold standard" diagnostic tool for anticipatory grief in the Thai context is not yet established.

Revision details: We have added this as a significant limitation in the Discussion section. We clarified that while the Thai PG-12-R demonstrates strong psychometric properties as a severity measure, establishing a clinical cut-off score through structured clinical interviews is a priority for our future research.

3. SEPARATING REGRESSION AND CORRELATION COEFFICIENTS: We agree that separating these results improves clarity for the reader.

Revision details: We have reorganized the tables as follows:

Table 3 now focuses on Spearman’s correlation coefficients.

Table 4 now focuses on Simple Linear Regression Analysis.

4. STRATIFYING TABLE 1 (DEMOGRAPHICS): This stratification provides a much clearer picture of the participant characteristics.

Revision details: We have updated Table 1 by stratifying the participants into "Low-score" and "High-score" groups based on the median score (17). We also included p-values to show demographic differences between these two groups.

### Responses to Journal Requirements & Reference List

Structural Reorganization: We have reorganized the Results section to present Validity findings (Face, Content, and Construct Validity) prior to Reliability (Internal Consistency and Test-Retest). This aligns with the logical progression of the Methods section.

Reference List: We have thoroughly reviewed the reference list. We have removed "Available from" and URL links for entries that have a DOI, following PLOS ONE’s preference for cleaner citations.

New Citations: We have added references for EFA methodology (Hair et al., 2010; Costello & Osborne, 2005) to support the added analysis.

Table Order: We have ensured all tables are cited in the text in chronological order:

Table 1: Participant characteristics (Stratified)

Table 2: Content Validity Index (CVI)

Table 3: Convergent Validity (Correlation)

Table 4: Simple Linear Regression

Table 5: Intraclass Correlation Coefficient (ICC)

We hope that these revisions adequately address the reviewers' concerns and that the manuscript is now suitable for publication in PLOS ONE.

Sincerely,

Arthit Chaithanasarn, M.D. Corresponding Author

Attachment Submitted filename: Response_to_Reviewers_auresp_2.pdf

# Peer review: Decision Letter 2

**DOI:** 10.1371/journal.pone.0343917.r005

**Authors:** Mohd Ismail Ibrahim

15 Feb 2026

Cross-cultural adaptation and psychometric properties study of Prolonged Grief Disorder Questionnaire (PG-12-R) for caregivers of terminal cancer patients, Thai version

PONE-D-25-55651R2

Dear Dr. Chaithanasarn,

We’re pleased to inform you that your manuscript has been judged scientifically suitable for publication and will be formally accepted for publication once it meets all outstanding technical requirements.

Within one week, you’ll receive an e-mail detailing the required amendments. When these have been addressed, you’ll receive a formal acceptance letter and your manuscript will be scheduled for publication.

An invoice will be generated when your article is formally accepted. Please note, if your institution has a publishing partnership with PLOS and your article meets the relevant criteria, all or part of your publication costs will be covered. Please make sure your user information is up-to-date by logging into Editorial Manager at Editorial Manager® and clicking the ‘Update My Information' link at the top of the page. For questions related to billing, please contact billing support.

If your institution or institutions have a press office, please notify them about your upcoming paper to help maximize its impact. If they’ll be preparing press materials, please inform our press team as soon as possible -- no later than 48 hours after receiving the formal acceptance. Your manuscript will remain under strict press embargo until 2 pm Eastern Time on the date of publication. For more information, please contact onepress@plos.org.

Kind regards,

Mohd Ismail Ibrahim, MCom.Med

Academic Editor

PLOS One

Additional Editor Comments (optional):

Reviewers' comments:

Reviewer's Responses to Questions

**Comments to the Author**

1. If the authors have adequately addressed your comments raised in a previous round of review and you feel that this manuscript is now acceptable for publication, you may indicate that here to bypass the “Comments to the Author” section, enter your conflict of interest statement in the “Confidential to Editor” section, and submit your "Accept" recommendation.

Reviewer #1: All comments have been addressed

Reviewer #2: All comments have been addressed

**********

2. Is the manuscript technically sound, and do the data support the conclusions?

The manuscript must describe a technically sound piece of scientific research with data that supports the conclusions. Experiments must have been conducted rigorously, with appropriate controls, replication, and sample sizes. The conclusions must be drawn appropriately based on the data presented.

Reviewer #1: Yes

Reviewer #2: Yes

**********

3. Has the statistical analysis been performed appropriately and rigorously?

Reviewer #1: Yes

Reviewer #2: Yes

**********

4. Have the authors made all data underlying the findings in their manuscript fully available?

The PLOS Data policy requires authors to make all data underlying the findings described in their manuscript fully available without restriction, with rare exception (please refer to the Data Availability Statement in the manuscript PDF file). The data should be provided as part of the manuscript or its supporting information, or deposited to a public repository. For example, in addition to summary statistics, the data points behind means, medians and variance measures should be available. If there are restrictions on publicly sharing data—e.g. participant privacy or use of data from a third party—those must be specified.

Reviewer #1: Yes

Reviewer #2: Yes

**********

5. Is the manuscript presented in an intelligible fashion and written in standard English?

PLOS ONE does not copyedit accepted manuscripts, so the language in submitted articles must be clear, correct, and unambiguous. Any typographical or grammatical errors should be corrected at revision, so please note any specific errors here.

Reviewer #1: Yes

Reviewer #2: Yes

**********

6. Review Comments to the Author

Please use the space provided to explain your answers to the questions above. You may also include additional comments for the author, including concerns about dual publication, research ethics, or publication ethics. (Please upload your review as an attachment if it exceeds 20,000 characters)

Reviewer #1: Thank you for clarifying the comments, now the manuscript is more informative and very clear for the readers.

Reviewer #2: (No Response)

**********

7. PLOS authors have the option to publish the peer review history of their article (what does this mean?). If published, this will include your full peer review and any attached files.

If you choose “no”, your identity will remain anonymous but your review may still be made public.

**Do you want your identity to be public for this peer review?**  For information about this choice, including consent withdrawal, please see our Privacy Policy.

Reviewer #1: No

Reviewer #2: No

**********

# Editor report: Acceptance letter

**DOI:** 10.1371/journal.pone.0343917.r006

**Authors:** Mohd Ismail Ibrahim

PONE-D-25-55651R2

PLOS One

Dear Dr. Chaithanasarn,

I'm pleased to inform you that your manuscript has been deemed suitable for publication in PLOS One. Congratulations! Your manuscript is now being handed over to our production team.

At this stage, our production department will prepare your paper for publication. This includes ensuring the following:

* All references, tables, and figures are properly cited

* All relevant supporting information is included in the manuscript submission,

* There are no issues that prevent the paper from being properly typeset

You will receive further instructions from the production team, including instructions on how to review your proof when it is ready. Please keep in mind that we are working through a large volume of accepted articles, so please give us a few days to review your paper and let you know the next and final steps.

Lastly, if your institution or institutions have a press office, please let them know about your upcoming paper now to help maximize its impact. If they'll be preparing press materials, please inform our press team within the next 48 hours. Your manuscript will remain under strict press embargo until 2 pm Eastern Time on the date of publication. For more information, please contact onepress@plos.org.

You will receive an invoice from PLOS for your publication fee after your manuscript has reached the completed accept phase. If you receive an email requesting payment before acceptance or for any other service, this may be a phishing scheme. Learn how to identify phishing emails and protect your accounts at https://explore.plos.org/phishing.

If we can help with anything else, please email us at customercare@plos.org.

Thank you for submitting your work to PLOS ONE and supporting open access.

Kind regards,

PLOS ONE Editorial Office Staff

on behalf of

Dr. Mohd Ismail Ibrahim

Academic Editor

PLOS One
